# Pandemic Poetry

**DOI:** 10.1177/1077800420948099

**Published:** 2020-08-08

**Authors:** Maria K. E. Lahman, Becky De Oliveira, Xiaoping Fan, James Hodges, Idilio Moncivais, Emily Royse

**Affiliations:** 1 University of Northern Colorado, Greeley, USA

**Keywords:** pandemic, COVID, poetry, research poetry

## Abstract

Members of a graduate educational ethnography course created pandemic poetry. The instructor was pedagogically stretched in translating what had always been an in-person research poetry creation experience to a virtual setting, but the students responded with powerful poetic renditions. In this article, course members’ poetry is featured, and a brief review of the course materials used and guidance provided to the class, along with a methodological reflection, are offered.

## An Irregular Ode to the Chair

A silky gray, blending with the velvety darkness yet standing strong against the golden blaze of the daytime.

Freckles of the metals you are mixed with dancing in the trickles of light shine through the blinds of the window—the blinds blocking the light to steal some of the joy of your embrace with the sun in a fit of jealousy.

Long, strong rods, adorned by sweat, gentle enough not to pierce soft, medium-Brown flesh, weakened by sedentary days but strong enough to endure the 300-plus pounds it weathers every day.

300-plus pounds of anxiety—the scribbles and tiny utterances of to-dos, what-ifs, hows, and whys. The mighty rods wrap in an embrace, a hug, a holding.

300-plus pounds of frustration. The staff says more cuts. More planning for the hypothetical.

Dry replies and check-ins in which nothing is said, but everything is said.

The chair is rocked back, but the solid foundation swings back with the rock.

The chair swings back but pushes forward. A fresh breath—to say, always keep going.

Reminding that forward is the only option.

300-plus pounds of laughter. Friends share sweet wine and memories.

Reminiscences turn to shared chuckles and bellows.

The laughter vibrates along the strong metal rods.

Laughing back.

When the bed folded under pressure and couldn’t handle the sleepless night,

the chair with its 24-hour sign was there.

It loved the blue haze of the laptop screen against the crisp dark room.

When the sofa couldn’t handle the rattles of restlessness,

the chair welcomed the hum of jittering legs and feet.

Oh, to be a chair in a global pandemic. The forgotten essential worker.

—*James Hodges*

Neighbors gathering around squad cars.

All lights are on. The place is loud.

The porch is a mess. The doors are open.

The cries of help can be heard through.

It is clear she was suffering.

The pain is visible in her face

The black eye, the broken lips.

The sad victim of domestic violence.

The stories are always the same:

Please officer make it stop!

It’s not his fault, he isn’t like that.

It was my fault. I should’ve known better.

Stop. Don’t rough him up, you brute!

Do not take him away. He is my man!

Who will bring the bacon, pay the bills?

Me and the kids will go crazy, please!

—*Idilio Moncivais*

## Distancing

Wearing a black

mask that keeps slipping

off my ears, I approach

the woman who waves

me over. *I would like*

*to buy a gun*, I say,

silently regretting the

phrasing. *I would not*

*like to buy a gun, but*

*I am compelled—like*

*someone is holding a*

*gun to my head.* I am

angry but making a

special effort to be

nice. Whose fault is

this, anyway?

She gives me over to

a man who is almost

eleven years to the day

my senior, a fun fact we

discover during my

background check. He

tells me, *I can’t give you*

*advice on what to answer.*

He tells me he can’t sell

me any ammo (what they

call bullets) today

because

people have been

stockpiling it for

weeks, buying

thousands of rounds,

enough to bring down

an army, maybe.

He draws an air rectangle

from his shoulders to his

hips and tells me this is

where I need to aim. *If*

*you shoot an intruder in*

*the leg, you will go to*

*jail.* He shows me the

ideal distance from which

to fire by pointing out

a man, just an ordinary

man in a surgical mask

standing idly by a table

perhaps seven yards away

and waiting for his turn.

God forbid I ever

have to fire this thing, I will

go ahead and aim for the leg,

and take whatever hell

they see fit to unleash. *They*

*cannot make me commit*

*a mortal sin*, I tell myself,

but my heart collapses under

the weight of those

words, because of course

they can. There is no

limit to what they can

make me do. I am here,

aren’t I? Eighty-five

minutes from start to

finish. And who are they

anyway? Legion, for we

are many.

—*Becky De Oliveira*

## Covid-Cute

“You look so cute.” I

adjust my daughter’s face mask—

and then I tremble.

What have I said? Covid-cute—

God forgive privilege—I can’t.

—*Maria Lahman*

## The School Gym

Coming to the gym,

Having the secret handshake

With the physical education teacher.

Engage in activities and games

With friends.

Cheering “Go, go, go,

You can go through it”

Everyone is sweaty and smiling.

Now everyone is at home.

Want to go to school

To see friends,

Joke with friends.

Now we keep social distance.

Really, really hope that

It is not as bad as I think it is going to get.

Hope I can back to the gym soon.

—*Xiaoping Fan*

## The Pandemic Wife

   *Do you even know how to sew?*

The pandemic wife channels the domestic ways of her elders,

Who at some point gifted her a padded green box

  printed with hearts, spools, and thimbles.

Inside, a menagerie of loose pins and lost treasures,

  buttons, fool’s gold, and scissors too small for her hands.

The last craft she abandoned lies in repose:

  a stick figure doll with pink fabric flesh,

  hand-sewn with crooked stitches still attached to a guiding needle.

Her stitches now are no straighter

As she rations small spools of mismatched thread

  to piece together cotton cloth, elastic bands, and floral wire.

Occasionally, she pierces her finger as she jabs the dull needle

  through gray pleats, speckled with red.

*How does it fit?*

She asks her masked spouse as he leaves for the office,

  as if she had the skill to change a thing.

  Tomorrow’s garment will be the same.

The pandemic wife channels the domestic ways of her elders,

Who at some point gifted her a child’s toolbox

  not meant for making personal protective equipment.

—*Emily Royse*

## Methodological and Pedagogical Note

With the rest of the nation’s academy, our face-to-face graduate course on educational ethnography shifted to virtual meetings over the course of a spring break. Amid the scramble to move all the students’ research to virtual only, amend institutional review boards (IRBs) as needed, figure out how to teach and learn with children at our feet (perhaps it should always be this way), and check in on one another’s welfare, I at first determined now was not the time to hold the course session on research poetry as I usually do. It would be too much cognitive dissonance—last time someone elected to sit out from the experience. How could I translate a warm, classroom face-to-face environment where students moved through centers at their own pace creating poetry while chatting with classmates to a virtual one?

Waking deep in the night with work on my mind as usual, I review my students, shuffling their images through my mind—are they safe, sound, and in a position to learn? James should be celebrating graduation and a birthday with a first-time sibling beach trip. All of Becky’s family members, including a university student son and commuter husband, are living and working back at home while she is also needing to prepare for comps. Ping, an ocean and half a country away from family and friends, is an ever-cheerful presence as if to say, this is always the lot of the international student. Emmy already had too much stress this semester with comps, orals, and migraines. Idilio, improbably, is a police officer *and* a doctoral student—a frontline person at all times and especially at this time.

I sharply pivot. What if at least one of the students is ready to think and express in poetic ways? What if poetry is *exactly* what someone needs in a time full of end-of-year comprehensive and stat examinations? I recall past poets from past research courses, including one who went on to publish research poetry prolifically. So we write, and then deeply moved I ask permission to publish since I know even the “princess problems” of academics are real—loneliness, debt, stress, depression, long-long hours, deep thoughts, and darkness at times.

  We create poetry,

  I am sure you agree we did—

  powerful poetry,

  in a time of pandemic.

### Poetry Course Instructions and Resources

What follows is a shortened version of the instructions and resources students were provided.

“*Share it with your enemies too*.” (Author unknown)

April is National Poetry Month in the United States.

There is a new module in Canvas on poetry.

If you wish, warm up for this experience by watching one of the following videos available in the module.

National Poetry Month #Poetry Defined https://www.youtube.com/watch?v=-GBH24zOxdc

What Is Poetry? #Poetry Defined https://www.youtube.com/watch?v=3fKCOb8qGQE

What Makes a Poem a Poem? Ted Ed https://www.youtube.com/watch?v=JwhouCNq-Fc

For this activity, you will either

1. Consider the pandemic and the experiences you are having with it as data

OR

2. Use observational field notes and interview transcripts as data.

Some of you may want to do all of the writing activities, but for class purposes you only need to choose one.

Each person will share one poem virtually during class in the form or way they wish.

Let me know if this causes anxiety in any way, and we can do break-out groups or figure out some other idea.

### Pandemic as Data

If you are considering writing about the pandemic, here are some phrases I am mulling over that might be a prompt for you.

Pandemic Poetry

Poetry in a Time of Plague—Pandemic Privilege—Pandemic Pedagogy

Here are experiences that might be a source of data:

- Being far from family or home during this time
- Being in a state/country that may not be handling that crisis as well as your state/country of origin is
- Moving face-to-face research to virtual
- Working as an essential worker
- Teaching children at home while working and going to school
- Having your whole family live at home who usually live elsewhere
- Feeling really down, worried, anxious, and helpless.
- Having loved ones be sick
- Working as a frontline responder and then relating to civilians
- Being middle class and having a solid income, home, and resources
- Not being middle class or having income loss and lack of resources
- Not having health insurance
- Having health insurance

Of course, there are many more.

### Poetry Choices

1. Free Verse

Watch the video on what is free verse. https://www.youtube.com/watch?v=lgM9wu3MaD0

#poetrydefined

- Read posted examples of free verse poetry

2. Ode

- The contemporary Ode is not required to have a form.
- American odes often point out the lofty in something that is humble such as Red Wing by Joseph Miller, an ode to work boots and thereby workers.
- Watch the video on what an ode is. #poetrydefined https://www.youtube.com/watch?v=rMmsMPNIn1g
- Watch the example of an ode by Clint Smith put to graphics by Ted Ed. https://www.youtube.com/watch?v=OGoehR_k0Xk

3. Haiku

- There are only three lines, totaling 17 syllables.
- The first line is five syllables.
- The second line is seven syllables.
- The third line is five syllables like the first.
- Punctuation and capitalization are up to the poet and need not follow the rigid rules used in structuring sentences.
- Watch Haiku by Poetry Defined https://www.youtube.com/watch?v=OUNc6L90UT8

Most importantly have fun!

### Love Is for Everybody: Final Class Period

Our final class period was held through Zoom. Along with reports on research studies, students shared the poetry they had created. For me, this is always a jaw-dropping experience as research poetry novices share their first ventures. We ended the semester by viewing the graphic video version of the poem “For Estefani” by Aracelis Girmay.

https://www.youtube.com/watch?v=DcIYMCC0M1w&list=PLJicmE8fK0Egxi0hgy5Tw-NFyLcpJ4bzJ&index=13&t=0s

Even more so than at the end of the semester, given the context of protest during a pandemic that has gripped the nation, if you have not seen the graphic video version of this poem I encourage you to take a look—Love is for everybody.

